# Colonic tuberculosis: a case report

**DOI:** 10.2144/fsoa-2022-0056

**Published:** 2023-02-27

**Authors:** Ghada Gharbi, Moufida Mahmoudi, Manel Yakoubi, Asma Ben Mohamed, Amal Khsiba, Mohamed Karim M'Farrej, Mahdi Bouassida, Emna Chelbi, Lamine Hamzaoui

**Affiliations:** 1 Department of Gastroenterology, Mohamed Taher Maamouri Hospital, Nabeul, Tunisia; 2 Department of Pathology, Mohamed Taher Maamouri Hospital, Nabeul, Tunisia; 3 Department of Surgery, Mohamed Taher Maamouri Hospital, Nabeul, Tunisia

**Keywords:** colonic tuberculosis, diagnosis, endoscopy, intestinal tuberculosis

## Abstract

**Aim:**

Colonic tuberculosis is rare. It accounts for 2–3% of abdominal tuberculosis. Clinical, radiological and endoscopic features are nonspecific. The diagnosis must be considered in front of chronic abdominal pain, vesperal fever and weight loss with on colonoscopy the presence of nodules or ulcers. The diagnosis is made on pathological findings.

**Case report:**

We report a case of an 82-year-old female patient with the diagnosis of colonic tuberculosis. The diagnosis were suspected on clinical presentation: chronic abdominal pain, fever and weight loss. The colonoscopy showed a nodular aspect of the left and sigmoid colonic mucosa and the pathology examination of the multiple biopsy specimens showed an epithelioid and gigantocellular granulomas with caseous necrosis.

**Conclusion:**

In front of a nonspecific clinical and endoscopic aspects, multiples colonic biopsies are mandatory to rule out differential diagnosis and confirm colonic tuberculosis.

## Plain language summary

Clinical and radiological features of colonic tuberculosis are nonspecific. The diagnosis must be considered in case of abdominal chronic pain and general symptoms. The mainly differential diagnosis are colorectal cancer or Crohn's disease. We report a case of an 82-year-old female patient with the diagnosis of colonic tuberculosis.

Tuberculosis remains a public health problem in developing countries. Abdominal tuberculosis accounts for 5% of all cases of tuberculosis [1] and colonic involvement is seen in 2–3% of patients with abdominal tuberculosis [2].

Clinical manifestations and endoscopic appearances of colonic tuberculosis are nonspecific. We report a case of an 82-year-old female patient with the diagnosis of colonic tuberculosis.

## Case report

An 82-year-old female patient, without significant medical or surgical history, consulted with the complaint of a 6-month history of diffuse abdominal pain, fever, loss of appetite and weight loss.

**Figure 1.**
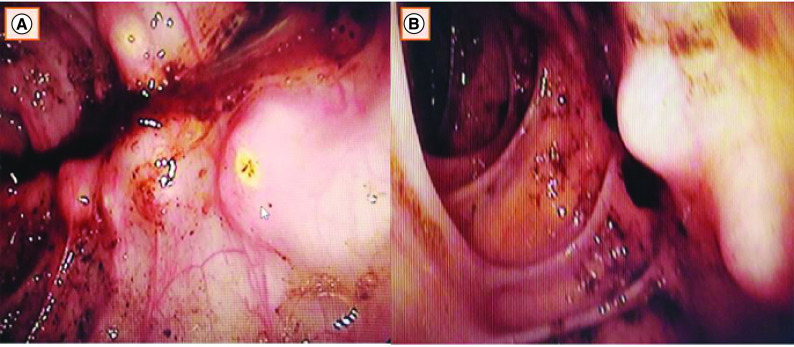
Colonoscopy found a nodular left and sigmoid colonic mucosa with the extrusion of a thick, whitish fluid when biopsied. **(A)** Nodular aspect of the left colonic mucosa on colonoscopy. **(B)** Nodules of 5–10 mm in diameter in the left colon.

The physical examination found a sub-febrile patient at 37.8°C. The abdominal examination was normal. Biological data showed microcytic hypochromic anemia of 10.6 g/dl, biological inflammatory syndrome (sedimentation rate: 80 mm, CRP: 89 mg/l). The albumin level was 28 g/l. The chest x-ray was normal. The abdominal CT scan noted a parietal thickening of the sigmoid colon. Colonoscopy found a nodular left and sigmoid colonic mucosa with the extrusion of a thick, whitish fluid when biopsied (Figure 1).

**Figure 2.**
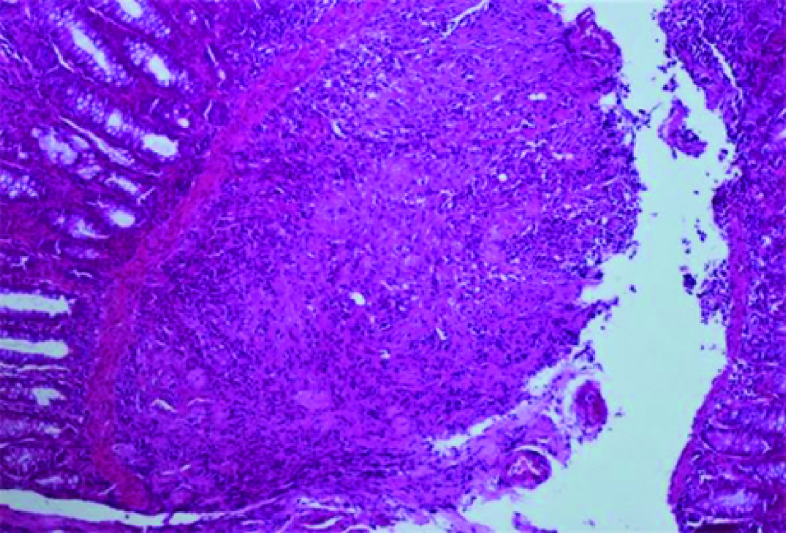
Pathological aspect of colonic mucosa. Numerous epitheloid granulomas in the submucosa (×100).

**Figure 3.**
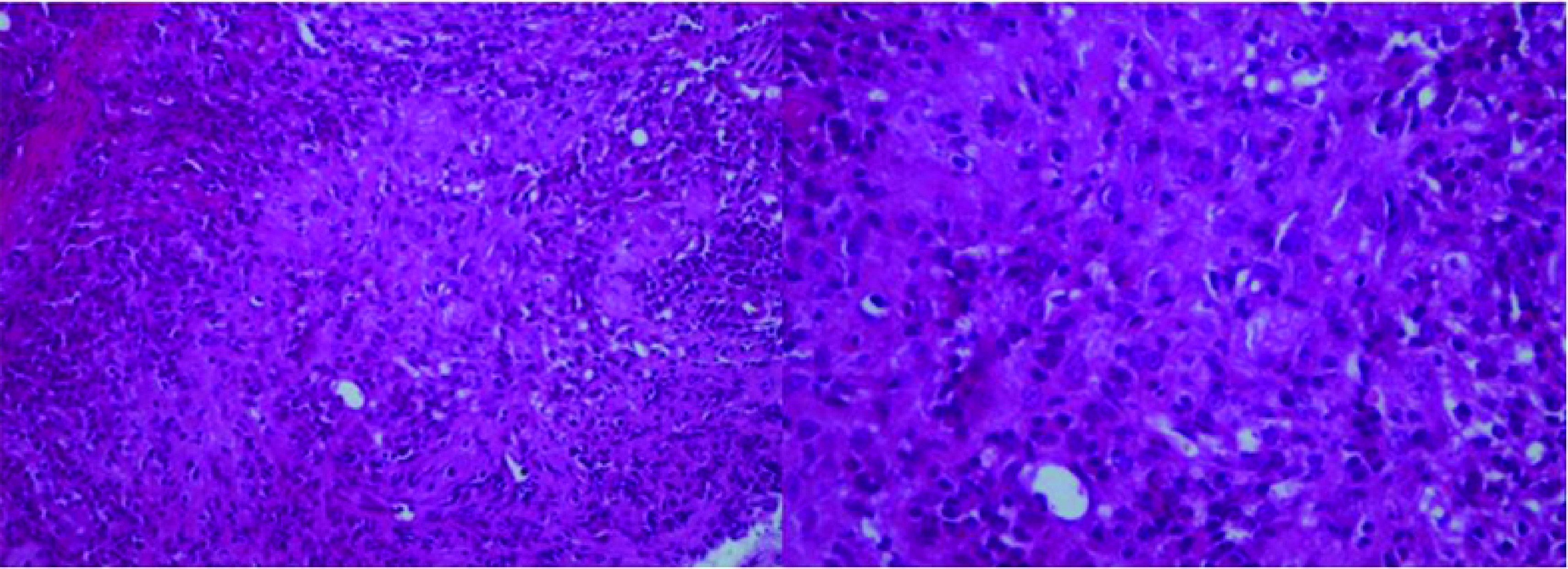
Epitheloid granuloma. Spindle-shaped or ovoid epitheloid cells. Absence of caseous necrosis (×200).

The pathology examination of the multiple biopsy specimens showed an epithelioid and gigantocellular granulomas with caseous necrosis (Figures 2 & 3). Thus, the patient was diagnosed with colonic tuberculosis. Anti-tuberculosis treatment was initiated. Since tuberculosis is endemic in developing countries, no specimens for special culture and sensitivities were required before treatment.

## Discussion

The incidence of extrapulmonary tuberculosis has increased in the last few years, even in developed countries, due to the increased incidence of HIV infection, making individuals vulnerable to tuberculosis [3].

In developing countries, it remains endemic despite the availability of an effective antituberculosis chemotherapy and pasteurized milk. In developed countries, the main risk factors are HIV infection, chronic renal disease and immunosuppression with prolonged steroid therapy [4].

Abdominal tuberculosis accounts for 5% of the cases of tuberculosis and 1–3% of them are gastrointestinal [5]. It mainly involves the ileocecal region and the adjoining part of the ascending colon [6]. Colorectal tuberculosis accounts for 11% of all gastrointestinal tuberculosis. The most commonly-affected sites are the transverse colon, the rectum, followed by the ascending colon [2].

Digestive involvement may be primary through direct ingestion of mycobacterium or secondary to highly bacilliferous lung lesions via the hematogenous or lymphatic route [7]. The bacterial agent is most often bovine or human Kokh's bacillus, exceptionally it is mycobacteria in immunocompromised subjects [7].

Clinical manifestations in colonic tuberculosis are less acute and less specific than the small intestine and the ileocecal localizations and therefore responsible for a delayed diagnosis [8]. Chronic abdominal pain is the most frequent presentation. Others manifestations are weight loss (80%), fever (66%), constipation (40%), ascites (40 to 100%), diarrhea (15%) with sometimes a dysenteric syndrome in the case of rectosigmoid location [9]. Sometimes, it can cause recurrent subacute intestinal obstruction episodes [6].

The most frequent colonoscopic features are transverse or linear ulcers or nodules [10]. Our patient had multiples nodules. Sometimes, a white fluid is extruded on biopsy [11], as it was the case in our patient.

Radiographic findings may help the diagnosis in case of pulmonary location but only quarter of patients with intestinal tuberculosis have evidence of active pulmonary tuberculosis [10].

Adenopathies are the most common site of extra-pulmonary tuberculosis. However, additional tests, such as physical examination and an abdominal CT did not detect any adenopathies in our patient. Searching of other sites isn't systematic.

The diagnosis is made on histopathological data showing granulomas with epitheloid cells and Langhans' giant cells with caseation and acid and alcohol-fast bacilli [12].

The presentation often mimics a colorectal cancer or Crohn's disease which are the mainly differential diagnosis [2,13]. Pathological data showing granulomas with caseation is specific to tuberculosis and eliminate other diagnosis.

The treatment is based on antituberculosis chemotherapy with usually a good response.

Surgery should be considered in case of complications such as intestinal obstruction (15–60%), fistula (25%), perforation (15%) and hemorrhage [14–16]. When perforation occurs, mortality approaches 30–40% [17].

Our case report emphasizes that multiple biopsy specimens and pathological data showing granulomas with caseation are necessary for the diagnosis of colonic tuberculosis in front of a colonic lesion. The main differntial diagnoses to rule out are neoplasia and Crohn's disease.

## Conclusion

Colonic tuberculosis is rare with nonspecific clinical, radiological and endoscopic features. However, the diagnosis must be considered in patients with chronic abdominal pain and weight loss.

**Summary points**

- Clinical and radiological features of colonic tuberculosis are nonspecific. The diagnosis must be considered in case of abdominal chronic pain and general symptoms.
- The mainly differential diagnosis are colorectal cancer or Crohn's disease.
- In front of a nonspecific clinical and endoscopic aspects, multiples colonic biopsies are mandatory to rule out differential diagnosis and confirm colonic tuberculosis.

## References

[1] Sharma SK, Mohan A. Extrapulmonary tuberculosis. Indian J. Med. Res. 120(4), 316–353 (2004). 15520485

[2] Sarma DR, Thebe PR, Bhardwaj R. Colonic tuberculosis masquerading as colorectal malignancy. Int. J. Colorectal. Dis. 30(7), 997–998 (2015). 25510815 10.1007/s00384-014-2092-7

[3] Mehta JB, Dutt A, Harvill L, Mathews KM. Epidemiology of extrapulmonary tuberculosis: a comparative analysis with pre-AIDS era. Chest 99(5), 1134–1138 (1999). 10.1378/chest.99.5.1134 2019168

[4] Sáenz EV, Magro PMH, Fernández JFÁ-T, Ovalle MV. Colonic tuberculosis. Dig. Dis. Sci. 47(9), 2045–2048 (2002). 12353853 10.1023/a:1019624913037

[5] Sheer TA, Coyle WJ. Gastrointestinal tuberculosis. Curr. Gastroenterol. Rep. 5(4), 273–278 (2003). 12864956 10.1007/s11894-003-0063-1

[6] Chaudhary A, Gupta NM. Colorectal tuberculosis. Dis. Colon Rectum 29(11), 738–741 (1986). 3769689 10.1007/BF02555322

[7] De Jesus LE, Marques AM, Rocha MS, Brom BRC, Siqueira RR. Left colon stenosis caused by tuberculosis. J. Pediatr. Surg., 39(10), 5–7 (2004). 10.1016/j.jpedsurg.2004.06.041 15486882

[8] Kaplanski G, Granel B, Payan MJ Fistulated sigmoid pseudodiverticulitis of tuberculous origin. Rev. Med. Interne 19(6), 447–448 (1998). 9775190 10.1016/s0248-8663(98)80873-4

[9] Foster BD, Buchberg B, Parekh NK, Mills S. Case of intestinal tuberculosis mimicking Crohn's disease. Am. J. Case. Rep. 13, 58–61 (2012). 23569489 10.12659/AJCR.882756 PMC3615969

[10] Misra SP, Misra V, Dwivedi M, Gupta SC. Colonic tuberculosis: clinical features, endoscopic appearance and management. J. Gastroenterol. Hepatol. 14(7), 723–729 (1999). 10440219 10.1046/j.1440-1746.1999.01940.x

[11] Al-Shamali Y, Al-Taweel T. Colonic tuberculosis. Gastrointest. Endosc. 84(5), 870–872 (2016). 26853297 10.1016/j.gie.2016.01.061

[12] Venables GS, Rana PS. Colonic tuberculosis. Postgrad. Med. J. 55(642), 276–278 (1979). 471865 10.1136/pgmj.55.642.276 PMC2428132

[13] Kedia S, Das P, Madhusudhan KS Differentiating Crohn's disease from intestinal tuberculosis. World J. Gastroenterol. 25(4), 418–432 (2019). 30700939 10.3748/wjg.v25.i4.418 PMC6350172

[14] Chen WS, Leu SY, Hsu H, Lin JK, Lin TC. Trend of large bowel tuberculosis and the relation with pulmonary tuberculosis. Dis. Colon Rectum 35(2), 189–192 (1992). 1735323 10.1007/BF02050677

[15] McGee GS, Williams LF, Potts J, Barnwell S, Sawyers JL. Gastrointestinal tuberculosis: resurgence of an old pathogen. Am. Surg. 55(1), 16–20 (1989). 2643908

[16] Horvath KD, Whelan RL. Intestinal tuberculosis: return of an old disease. Am. J. Gastroenterol. 93(5), 692–696 (1998). 9625110 10.1111/j.1572-0241.1998.207_a.x

[17] Kapoor VK. Abdominal tuberculosis. Postgrad. Med. J. 74(874), 459–467 (1998). 9926119 10.1136/pgmj.74.874.459 PMC2360888

